# The role of serum metrics in anti- VEGF treatment for macular edema induced by retinal vein occlusion

**DOI:** 10.1186/s12886-023-02921-5

**Published:** 2023-04-24

**Authors:** Yun-Chang Wang, Chuan-Qi Zhou, Rong-Rong Li, Yi Cai, Meng-Meng Wang, Li-Fei Yuan, Yi-Qing He

**Affiliations:** Heibei Eye Hospital, Number 399 quan bei dong da jie, Xingtai, Hebei Province 054001 China

**Keywords:** Retinal vein occlusion, Macular edema, Anti-VEGF treatment, Platelets, Platelet-to-lymphocyte ratio ( PLR)

## Abstract

**Aim:**

To evaluate association between pretreatment serum metrics and best corrected visual acuity ( BCVA) of patients with macular edema secondary to retinal vein occlusion and its subtypes after intravitreal ranibizumab or conbercept implant.

**Methods:**

This prospective research included 201 patients(201 eyes) who were diagnosed with macular edema secondary to retinal vein occlusion at Heibei Eye Hospital between January 2020 and January 2021, who all received intravitreal anti- vascular endothelial growth factor treatment. Serum metrics were measured before the first treatment, and correlations between BCVA and each of four parameters— platelets, neutrophil- to- lymphocyte ratio(NLR), platelet- to- lymphocyte ratio(PLR) and monocyte- to- lymphocyte ratio(MLR)— were analyzed to identify predictors of effective intravitreal injection treatment outcomes.

**Results:**

The mean platelets was significantly different in the effective and ineffective group for RVO-ME (273.02 ± 41.49 × 109/L,214.54 ± 44.08 × 109/L P < 0.01),BRVO-ME (269.43 ± 49.52 × 109/L,214.72 ± 40.42 × 109/L P < 0.01), and CRVO-ME (262.32 ± 32.41 × 109/L,209.27 ± 42 0.91 × 109/L P < 0.01). The cutoff value of the platelets was 266.500, the area under the curve was 0.857,and the sensitivity and specificity were 59.8% and 93.6%, respectively. The mean PLR was significantly different in the effective and ineffective group for RVO-ME (154.66 ± 49.60, 122.77± 44.63 P < 0.01),BRVO-ME (152.24 ± 54.99, 124.72 ± 41.46 P = 0.003), and CRVO-ME (152.06±44.23, 118.67 ± 41.80 P = 0.001). The cutoff value of the platelets was 126.734, the area under the curve was 0.699, and the sensitivity and specificity were 70.7% and 63.3%, respectively. There were no statistical differencies between the effective and ineffective group(RVO- ME and its subtypes) in NLR and MLR.

**Conclusion:**

Higher pretreatment platelets and PLR were associated with BCVA in patients with RVO- ME and its subtypes who were treated with anti- VEGF drugs. The platelets and PLR may be used as predictive and prognostic tools for effective intravitreal injection treatment outcomes.

## Introduction

Retinal vein occlusion (RVO) is the second most common retinal vascular disorder after diabetic retinopathy. Retinal ischemia and hypoxia may be present due to RVO, and the increase of vascular permeability leads to sub-or intra-retinal exudation, which results in the appearance of macular edema (ME). ME is a severe complication that can cause dramatic vision loss [1]. The exact pathological mechanism of thrombosis remains unclear. Hypertension, diabetes, cardiovascular and cerebrovascular diseases, hyperlipidemia, pregnancy, oral contraceptives, hypercoagulable state, and hyperhomocysteinemia have been found to be high-risk factors for RVO [2]. Furthermore, local and systemic inflammation can induce vascular sclerosis and hypercoagulable state, which accelerate the formation of venous thrombosis.

Vascular endothelial growth factor (VEGF) is a cytokine that regulates vascular permeability and endothelial cell mitosis; the tight junctions between retinal vascular endothelial cells will be phosphorylated, and their content can be changed [3]. Anti-VEGF therapy can reduce the leakage and prevent the remodeling of blood vessels, thereby reducing ME. Compared to grid laser photocoagulation, anti-VEGF therapy is considered the first-line treatment for RVO-ME.

Rezar-Dreindl et al. proved that VEGF, IL-6, and IL-1 β are correlated with RVO-ME [4]. Moreover, several studies have shown that inflammatory factors play an important role in RVO-ME. The prognoses of malignant tumors and cardiovascular abnormal infectious diseases have been confirmed to be correlated with NLR, MLR, and PLR [5]; however, the correlations between NLR, MLR, and PLR and the efficiency of anti-VEGF for RVO-ME have rarely been reported. This study will focus on these correlations and whether they can be indicators for the prognosis of RVO-ME.

## Subjects and methods

### Participants

From January 2020 to January 2021, 201 patients (201 eyes) were diagnosed with RVO-ME using fundus fluorescein angiography (FFA) and optical coherence tomography (OCT). The inclusion criteria were as follows: the patients (1) underwent a complete examination, including best-corrected visual acuity (BCVA), intraocular pressure (IOP), slit-lamp biomicroscopy, and fundus pre-set lens; (2) had RVO diagnosed with FFA and ME diagnosed with OCT; (3) voluntarily participated in the trial, signed the informed consent form, and completed the treatment and follow-up; and (4) had IOP in the normal range (10–21 mmHg). The exclusion criteria comprised the following: (1) other treatments for RVO-ME during the follow-up period, such as retinal laser photocoagulation, intravitreal corticosteroids injection, and corticosteroids retrobulbar or peribulbar injection; (2) patients with other concomitant ocular diseases, such as uveitis, age-related macular degeneration, glaucoma, and diabetes retinopathy, and other severe refracting opacity–affected examinees; (3) the presence of diabetes mellitus, systemic inflammatory diseases, cardiovascular diseases, sepsis, and malignant tumors; (4) a history of routine anti-inflammatory and anticoagulant drugs or oral contraceptives; (5) a history of alcoholism and heavy smoking; (6) a history of intraocular surgery, vitreous hemorrhage, endophthalmitis, ocular trauma, or other laser treatments.

### Methods

Two anti-VEGF drugs were used in this experiment: ranibizumab and conbercept. Everyone chose either of the anti-VEGF drugs on their own will randomly at the first treament for comparison, receiving intravitreal injections of 0.5 mg or 0.05 ml under aseptic conditions. The patients were divided into the efficient group and the inefficient group based on the BCVA results after treatment; those with better results were placed in the efficient group, and the others were placed in the inefficient group. The criteria for the efficient group was one line of improvement as minimal after treatment at least. If multiple injections of anti-VEGF drugs had been needed,the initial species was used for the following injections, only the results of the first treatment were analyzed. The patients returned for follow-ups 1 week postoperatively; they were then evaluated on a monthly basis and received injections as needed or pro re nata.

Blood samples were obtained from the antecubital vein before treatment at 6: 00 am. Leukocyte subtypes (neutrophils, lymphocytes, and monocytes) and platelets were measured using an Automated Blood Coagulation Analyzer (Sysmex XN-550, Japan). Afterward, the neutrophil-to-lymphocyte ratio (NLR), monocyte-to-lymphocyte ratio (MLR), and platelet-to-lymphocyte ratio (PLR) were calculated and recorded for all blood samples. The patients and their families signed informed consent forms, and this study was approved by the Medical Ethics Committee of Hebei Eye Hospital.

### Statistical analysis

All statistical analyses were conducted using SPSS 23.0 (SPSS, Inc., Chicago, IL, USA). Quantitative data were presented as mean ± standard deviation (SD). The independent samples t-test was used to compare the two sets of parametric data of each group. The categorical variables were expressed as percentages, which were compared using chi-square tests. Spearman’ s correlation analysis was used to analyze the correlation between the parameters. The optimal cutoffpoints of NLR, PLR, and MLR were determined using receiver operator characteristic (ROC) curve analysis. The areas under the curve (AUC) were calculated to determine the accuracy of the tests. A p-value less than 0.05 was considered significant in all of the analyses.

## Results

### Total results

There were no differences in the gender, age, RVO subtypes, anti-VEGF drugs, baseline BCVA, IOP before and after treatment, or neutrophil, lymphocyte, and monocyte counts of the patients in the efficient and inefficient groups. The differences in the changes in BCVA between the efficient and inefficient groups were remarkable (0.26 ± 0.22 vs. 0.09 ± 0.09, *P* < 0.01), but no significant differences in IOP were observed between the efficient and inefficient groups (3.67 ± 0.63 vs. 3.10 ± 0.58, *P* > 0.05). The platelet levels in the efficient group were higher than those in the inefficient group (273.02 ± 41.49 vs. 214.54 ± 44.08, *P* < 0.01). The PLR values in the efficient group were significantly higher than those in the inefficient group (154.66 ± 49.60 vs.

122.77 ± 44.63, *P <* 0.05). There were no differences in the NLR and MLR between the efficient and inefficient groups (2.01 ± 0.87 vs. 2.08 ± 1.08, *P >* 0.05 and 0.21 ± 0.07 vs. 0.23 ± 0.09, *P >* 0.05, respectively). The total results are shown in Table 1.

**Table 1:** Total results

	Inefficient group (n=109)	Efficient group index (n=92)	Statistic	*P*
**Baseline index**				
Age (year)	57.34 ± 9.42	57.07 ± 10.59	37.405(c^2^)	0.748
Male/female	55/54	45/47	0.008(c^2^)	0.929
BRVO/CRVO	65/44	50/42	0.360(c^2^)	0.548
Ranibizumab/Conbercept	66/43	57/35	0.392(c^2^)	0.531
BCVA at baseline (LogMAR)	0.73 ± 0.22	0.77 ± 0.37	-8.674(*t*)	0.544
BCVA after treatment (LogMAR)	0.82 ± 0.22	0.52 ± 0.26	4.526(*t*)	0.001
Change in BCVA (LogMAR)	0.09 ± 0.09	0.26 ± 0.22	-21.116(*t*)	*< 0.01*
IOP at baseline (mmHg)	16.24 ± 3.08	16.26 ± 3.64	0.043(*t*)	0.966
IOP after treatment (mmHg)	16.82 ± 2.83	16.89 ± 2.67	0.192(*t*)	0.848
Change in IOP (mmHg)	3.10 ± 0.58	3.67 ± 0.63	0.114(*t*)	0.909
**Laboratory results**				
White blood cells (x10^9^/L)	6.01 ± 1.48	6.13 ± 1.86	0.517(*t*)	0.606
Neutrophils (x10^9^/L)	3.62 ± 1.29	3.66 ± 1.46	0.187(*t*)	0.852
Monocytes (x10^9^/L)	0.41 ± 0.15	0.39 ± 0.13	-1.074(*t*)	0.284
Platelets (x10^9^/L)	214.54 ± 44.08	273.02 ± 41.49	9.625(*t*)	*< 0.01*
Lymphocytes (x10^9^/L)	1.91 ± 0.59	1.93 ± 0.61	0.273(*t*)	0.785
NLR	2.08 ± 1.08	2.01 ± 0.87	-0.530(*t*)	0.596
MLR	0.23 ± 0.09	0.21 ± 0.07	-1.475(*t*)	0.142
PLR	122.77 ± 44.63	154.66 ± 49.60	4.797(*t*)	*< 0.01*

### The results of the BRVO and CRVO groups

There were no differences in the gender, age, anti-VEGF drugs, baseline BCVA, IOP before and after treatment, or neutrophil, lymphocyte, and monocyte counts of the patients in the BRVO and CRVO groups. For BRVO, the platelet levels and PLR values in the efficient group were higher than those in the inefficient group (269.43 ± 49.52 vs. 214.72 ± 40.42, *P* < 0.01 and 152.24 ± 54.99 vs. 124.72 ± 41.46, *P* = 0.003, respectively). However, there were no statical differences in NLR or MLR between the efficient and inefficient groups (2.00 ± 0.78 vs. 1.98 ± 0.90, *P* > 0.05 and 0.21 ± 0.07 vs. 0.23 ± 0.07, *P* > 0.05). For CRVO, the platelet levels and PLR values in the efficient group were higher than those in the inefficient group (262.32 ± 32.41 vs. 209.27 ± 42.91, *P < 0 0.01* and 152.06 ± 44.23 vs. 118.67 ± 41.80, *P* = 0.001, respectively); however, there were no statical differences in NLR or MLR between the efficient and inefficient groups (2.00 ± 0.98 vs. 2.18 ± 1.17, *P* > 0.05 and 0.21 ± 0.08 vs. 0.24 ± 0.11, *P* > 0.05, respectively). The results of the BRVO and CRVO groups are shown in Tables 2 and 3 respectively.

**Table 2:** The BRVO groups

	Inefficient group (n = 65)	Efficient group (n = 50)	Statistic index	*P*
**Baseline index**				
Age (year)	56.42 ± 10.21	56.78 ± 9.35	33.638(c^2^)	0.627
Male/female	34/31	25/25	0.124(c^2^)	0.725
Ranibizumab/conbercept	38/27	31/19	0.030(c^2^)	0.863
BCVA at baseline (LogMAR)	0.59 ± 0.12	0.63 ± 0.35	5.094(*t*)	0.280
BCVA after treatment (LogMAR)	0.68 ± 0.14	0.44 ± 0.25	-6.647(*t*)	0.004
Change in BCVA (LogMAR)	0.09 ± 0.10	0.20 ± 0.12	-18.635(*t*)	*< 0.01*
IOP at baseline (mmHg)	16.12 ± 3.06	16.28 ± 3.32	0.271(*t*)	0.787
IOP after treatment (mmHg)	16.52 ± 2.97	16.94 ± 2.57	0.798(*t*)	0.427
Change in IOP (mmHg)	2.96 ± 0.40	3.67 ± 0.66	0.417(*t*)	0.677
**Laboratory results**				
White blood cells (x10^9^/L)	5.85 ± 1.50	6.27 ± 1.94	1.309(*t*)	0.193
Neutrophils (x10^9^/L)	3.45 ± 1.17	3.72 ± 1.44	1.108(*t*)	0.270
Monocytes (x10^9^/L)	0.40 ± 0.12	0.40 ± 0.15	-0.058(*t*)	0.954
Platelets (x10^9^/L)	214.72 ± 40.42	269.43 ± 49.52	6.552(*t*)	*< 0.01*
Lymphocytes (x10^9^/L)	1.86 ± 0.55	1.95 ± 0.60	0.803(*t*)	0.424
NLR	1.98 ± 0.90	2.00 ± 0.78	0.112(*t*)	0.911
MLR	0.23 ± 0.07	0.21 ± 0.07	-1.062(*t*)	0.290
PLR	124.72 ± 41.46	152.24 ± 54.99	3.073(*t*)	0.003

**Table 3:** The CRVO groups

	Inefficient group (n = 44)	Efficient group (n = 42)	Statistic index	*P*
**Baseline index**				
Age (year)	57.03 ± 11.03	57.34 ± 9.08	32.552(c^2^)	0.390
Male/female	21/23	20/22	0.009(c^2^)	0.923
Ranibizumab/conbercept	28/16	26/16	0.185(c^2^)	0.667
BCVA at baseline (LogMAR)	0.73 ± 0.22	0.78 ± 0.37	1.777(*t*)	0.081
BCVA after treatment (LogMAR)	0.82 ± 0.22	0.52 ± 0.26	-8.927(*t*)	0.001
Change in BCVA (LogMAR)	0.09 ± 0.07	0.25 ± 0.27	-11.720(*t*)	*<* 0.01
IOP at baseline (mmHg)	16.24 ± 3.08	16.26 ± 3.64	-0.378(*t*)	0.707
IOP after treatment (mmHg)	16.82 ± 2.83	16.89 ± 2.67	-0.570(*t*)	0.570
Change in IOP (mmHg)	3.23 ± 0.64	3.71 ± 0.60	-0.045(*t*)	0.964
**Laboratory results**				
White blood cells (x10^9^/L)	6.07 ± 1.42	5.85 ± 1.64	-0.651(*t*)	0.517
Neutrophils (x10^9^/L)	3.77 ± 1.41	3.47 ± 1.39	-0.978(*t*)	0.331
Monocytes (x10^9^/L)	0.43 ± 0.19	0.37 ± 0.11	-1.782(*t*)	0.079
Platelets (x10^9^/L)	209.27 ± 42.91	262.32 ± 32.41	6.317(*t*)	*< 0.01*
Lymphocytes (x10^9^/L)	1.91 ± 0.61	1.87 ± 0.57	-0.375(*t*)	0.709
NLR	2.18 ± 1.17	2.00 ± 0.98	-0.794(*t*)	0.430
MLR	0.24 ± 0.11	0.21 ± 0.08	-1.386(*t*)	0.170
PLR	118.67 ± 41.80	152.06 ± 44.23	3.513(*t*)	0.001

### Ranibizumab and conbercept in the efficient groups of RVO and its subtypes

There were no differences in the NLR, MLR, PLR, or platelet levels of the two drugs in the efficient groups of RVO and its subtypes. The results are shown in Table 4.

**Table 4:** Ranibizumab and conbercept in the efficient groups of RVO and its subtypes

	RVO			CRVO			BRVO		
	Ranibizumab	Conbercept	*P*	Ranibizumab	Conbercept	*P*	Ranibizumab	Conbercept	*P*
	(n = 57)	(n = 35)		(n = 26)	(n = 16)		(n = 31)	(n = 19)	
Male/female	28/29	17/18	0.401	12/14	8/8	0.667	16/15	9/10	0.977
White blood cells (x10^9^/L)	6.14 ± 1.66	5.95 ± 1.66	0.442	5.89 ± 1.43	6.09 ± 1.71	0.565	6.07 ± 1.85	5.99 ± 1.53	0.793
Neutrophils (x10^9^/L)	3.64 ± 1.23	3.63 ± 1.56	0.964	3.53 ± 1.07	3.79 ± 1.84	0.491	3.55 ± 1.38	3.61 ± 1.19	0.808
Monocytes (x10^9^/L)	0.40 ± 0.13	0.40 ± 0.16	0.802	0.38 ± 0.14	0.43 ± 0.18	0.155	0.41 ± 0.14	0.39 ± 0.14	0.568
Platelets (x10^9^/L)	243.97 ± 47.34	237.20 ± 58.15	0.367	236.42 ± 47.62	234.70 ± 44.59	0.872	241.36 ± 56.51	235.24 ± 45.84	0.535
Lymphocytes (x10^9^/L)	1.96 ± 0.65	1.85 ± 0.50	0.200	1.84 ± 0.65	1.97 ± 0.47	0.335	1.95 ± 0.61	1.83 ± 0.51	0.285
NLR	2.00 ± 0.85	2.11 ± 1.17	0.461	2.13 ± 1.05	2.02 ± 1.13	0.644	1.92 ± 0.83	2.09 ± 0.87	0.281
MLR	0.22 ± 0.07	0.23 ± 0.09	0.398	0.22 ± 0.10	0.23 ± 0.09	0.851	0.22 ± 0.06	0.22 ± 0.07	0.822
PLR	137.36 ± 49.84	137.38 ± 49.25	0.998	140.93 ± 49.08	125.73 ± 38.83	0.150	135.87 ± 50.93	138.11 ± 48.20	0.811

### ROC curve

**Fig. 1:**
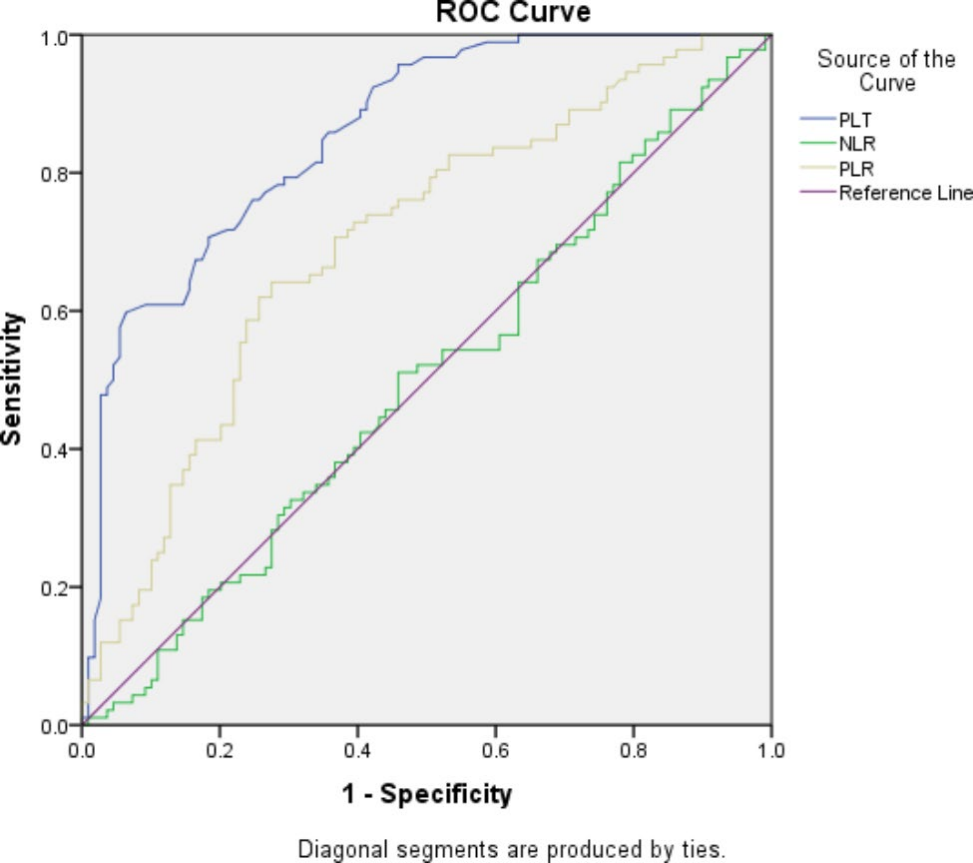
The ROC curve of platelets, NLR, and PLR

Platelets, PLR, and NLR were analyzed using the ROC curve. The optimal cutoff value of platelets was 266.500, with 59.8% sensitivity and 93.6% specificity (AUC: 0.857; 95% confidence interval [CI]: 0.807–0.908). The optimal cutoff value of PLR was 126.734, with 70.7% sensitivity and 63.3% specificity (AUC: 0.699; 95% CI: 0.627–0.772). The optimal cutoff value of NLR was 1.651, with 56.5% sensitivity and 36.7% specificity (AUC: 0.497; 95% CI: 0.417–0.577). The results are shown in Table 5; Fig. 1.

**Table 5:** The ROC parameters of platelets, NLR, and PLR

	AUC	95%CI	Sensitivity%	Specificity%
NLR (> 1.651)	0.497	0.417–0.577	56.5	36.7
PLR (> 126.734)	0.699	0.627–0.772	70.7	63.3
Platelets > 266.500	0.857	0.807–0.908	59.8	93.6

## Discussion

The correlations between the BCVA of RVO-ME after anti-VEGF therapies and platelets, PLR, NLR, and MLR have been presented in this study. The differences in the platelets and PLR of RVO and its subtypes between the efficient and inefficient groups were prominent after treatment; however, the outcomes of MLR and NLR were converse. Compared to NLR and MLR, platelets and PLR exhibited higher sensitivity and specificity. Hence, platelets and PLR can be used as an index to evaluate the prognosis of RVO-ME. However, this is not completely consistent with Rao’s conclusion [6].

Ray et al. showed that the inflammatory action mediated by leukocytes and its subtypes is closely related to the occurrence and development of tumors, as well as cardiovascular and cerebrovascular diseases [7, 8]; furthermore, the incidence rate and mortality of coronary heart disease are directly proportional to the level of neutrophils, and low-level lymphocytes are high-risk factors for coronary vascular disease [9]. NLR has been widely used to evaluate the incidence rate and prognosis of systemic inflammatory diseases [10]. It was confirmed that NLR in patients with RVO-ME was significantly higher compared to the control group byAhin [11], Ayhan [12] and Emrullah [13]. It was speculated that NLR could be used as a predictor of the occurrence and development of RVO-ME. However, in the studies of Antonio [14] and Esra [15], there was no significant difference in NLR between the RVO-ME group and the control group.

Platelets can regulate inflammatory reaction and the release of VEGF by interacting with neutrophils and vascular endothelial cells [16]. Activated neutrophils can release a variety of cytokines, including VEGF, which may damage the blood retinal barrier, increase the permeability of the vascular wall, and aggravate ME [6]. Therefore, platelets can indirectly lead to the occurrence of ME. Meanwhile, the increase of platelets is an important factor in the formation and development of retinal vein thrombosis [14]. Bian [17] confirmed that the platelet levels in the RVO group was significantly higher than those in the control group.

There was a positive correlation between platelet level, PLR, and VEGF level in the RVO-ME group at baseline [18]. From our research, it can be concluded that the BCVA outcome of the patients who underwent anti-VEGF treatment was significantly correlated with the platelet level and PLR at baseline. Thus, platelets and PLR can be used as independent predictors of the prognosis. However, there was no significant difference in MLR between the effective and ineffective groups. The number of lymphocytes and monocytes will decrease under the condition of increased systemic pressure and sub-health, which can be seen as a response to stress, but neither of these can regulate the release of VEGF [9]. Thus, MLR cannot be used as an independent predictor of the prognosis. However, Li [19] had arrived at a different conclusion, finding that MLR was significantly higher in the efficient group than in the inefficient group. This will be demonstrated in subsequent research.

Two anti-VEGF drugs were used in this study. Ranibizumab is a human anti-VEGF monoclonal antibody that can be used in combination with all VEGF-A active isomers. Conbercept is a humanized, soluble VEGF receptor (VEGFR) protein comprising extra-cellular domain-2 of VEGFR-1 and extra-cellular domain-3 and − 4 of VEGFR-2; all these domains are connected via the Fc region of human immunoglobulin G [20, 21]. These two anti-VEGF drugs can reduce the permeability of the vascular wall, promote the absorption of intraretinal and subretinal fluids, and improve the density of superficial and deep capillaries of the retina of posterior especially in macular.Furthermore, the function of the photoreceptor cells and ganglion cells of the macular will be further improved.

There were no significant differences regarding platelets, PLR, NLR, and MLR in the RVO group and its subtypes, and no significant differences were observed in the outcomes of the conbercept and ranibizumab groups.

This study has some limitations. First, recent occurrences of acute coronary artery disease and stroke, which will affect neutrophils, lymphocytes, and platelets, were not taken into account. Second, BCVA was the only index used as a predictor of the effectiveness of the treatment, and the changes in central macular thickness were not considered. Third, the interactions of other serum inflammatory factors, such as C-reactive protein, interleukin, and the tumor necrosis factor family, with platelets, neutrophils, and lymphocytes were not studied.

## Conclusion

In general, compared with other indexes (such as MPV, PCT, and PDW), PLR and platelets are cheap and convenient indexes for predicting the prognosis of the anti-VEGF treatment of RVO-ME. The role of NLR should be further demonstrated in the follow-up study.

## Data Availability

The datasets used and/or analyzed during the current study are available from the corresponding author on reasonable request.

## Abbreviations

BRVO: Branch retinal vein occlusion
CRVO: Central retinal vein occlusion
BCVA: Best-corrected visual acuity
IOP: Intraocular pressure
NLR: Neutrophil-to-lymphocyte ratio
MLR: Monocyte-to-lymphocyte ratio
PLR: Platelet-to-lymphocyte ratio
ROC: Receiver operating characteristic AUC:Area under curve
AUC: Area under curve
CI: Confidence level
